# Supplementary material for: Neural substrates of parallel devaluation-sensitive and devaluation-insensitive Pavlovian learning in humans
Source: Nat Commun. 2023 Dec 5;14:8057. doi: 10.1038/s41467-023-43747-5 (PMC10697955; doi:10.1038/s41467-023-43747-5)
Supplement: Supplementary file 1 — Supplementary Information [file 41467_2023_43747_MOESM1_ESM.pdf]

Supplementary information for:

Neural substrates of parallel devaluation-sensitive and devaluation-insensitive Pavlovian learning in humans

Pool, E. R., Pauli, W. M., Cross, L. and O'Doherty, J. P.

Supplementary Notes: Control and additional analyses

Pleasantness of the sweet and salty outcome

We tested the effect of the outcome's identity (sweet or salty) in interaction with the experimental run (first, second, or third) on the pleasantness ratings. We did not find any statistically significant result, and in particular, we did not find evidence suggesting a statistically significant difference in pleasantness between the salty and the sweet outcomes ($\beta = -0.270$, $SE = 0.294$, 95% CI $= [-0.847, 0.307]$, $p = 0.361$, $BF_{10} = 0.168$; see Sup. Fig. 1).

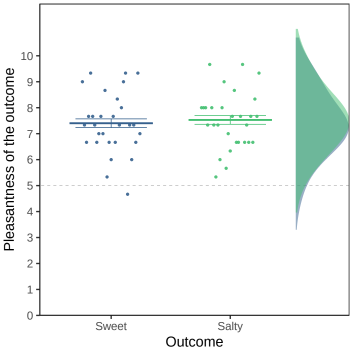

Supplementary Fig. 1 Pleasantness of the sweet and salty snack. Mean pleasantness ratings of sweet and salty snacks over the three runs of the experiment. Error bars indicate the within–participant s.e.m. $N = 29$ participants.

Pupil dilation and model-based value regressors

As a control analysis, we tested how well the two model-based regressors explained the pupil dilation responses to the CSs. We first derived the value regressor from the Rescorla-Wagner model and the Forward model using the best fitting learning rate for each participant. We then entered the value based regressor as within-participants fixed factor. As random effects, we modeled

random intercepts for participants (ID) and by-participant random slopes for the value regressor. The final models were built as follows (in lme4 syntax):

$$pupil \sim value + (1 + value \mid ID) \quad (1)$$

This analysis revealed that the value regressor derived from the Forward model significantly explains the pupil responses ($\beta = 0.062$, $SE = 0.024$, 95% CI = $[0.0150, 0.109]$, $p = 0.015$, $BF_{10} = 4.05$), while the regressor derived from the Rescorla-Wagner did not reach statistical significance ($\beta = 0.087$, $SE = 0.042$, 95% CI = $[0.003, 0.1710]$, $p = 0.053$, $BF_{10} = 2.52$).

Control analysis for the Pavlovian predictions about spatial location and taste identity attributes of the outcome

We ran this analysis as a sanity check of the primary analysis we used to identify the ROIs involved in the predictions of spatial location and taste identity attributes of the outcome. We used a different training and testing approach, which involved training and testing different runs to see if we could successfully decode the different outcome predictions on the ROIs we identified in the main analysis. Specifically, we divided the data in three folds, one per run. We trained the classifier to decode the CSs left vs. CSs right on two runs and then, tested the remaining one in a 3-Folds cross-validation. We used the same procedure to train the classifier to decode the CSs sweet vs the CSs salty. Using this approach, we could decode the identity in the right intraparietal sulcus (IPS; $ACC = 0.526$, $SE = 0.007$, 95% CI = $[0.511, 0.540]$, $p = 0.0008$, $BF_{10} = 39.028$), the post central gyrus (PCG; $ACC = 0.521$, $SE = 0.010$, 95% CI = $[0.500, 0.542]$, $p = 0.049$, $BF_{10} = 1.223$), and the paracentral lobule (PCL; $ACC = 0.525$, $SE = 0.010$, 95% CI = $[0.503, 0.547]$, $p = 0.025$, $BF_{10} =$

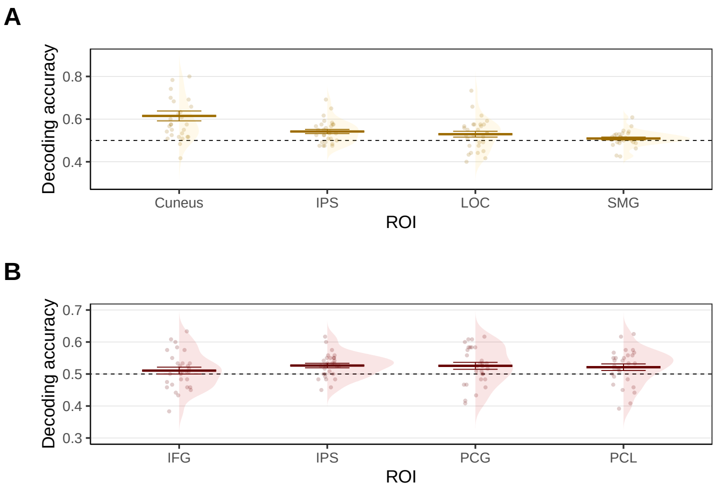

Supplementary Fig. 2 Mean classifier accuracies for the outcome identity **(A)** and the outcome side delivery **(B)** in the regions of interest (ROIs) from the main MVPA analysis, which cover parts of the intraparietal sulcus (IPS), the supra marginal gyrus (SMG), the lateral occipital complex (LOC), the inferior frontal gyrus (IFG), the superior temporal lobule and paracentral lobule (PCL) and the post central gyrus (PGC). Error bars represent 95% confidence interval adjusted for within participants designs. $N = 29$ participants.

2.060), but not in the right Inferior Frontal Gyrus (IFG; $ACC = 0.510$, $SE = 0.010$, 95% CI = $[0.488, 0.532]$, $p = 0.330$, $BF_{10} = 0.308$). We could decode the side in the ROIs covering parts of the Cuneus ($ACC = 0.614$, $SE = 0.023$, 95% CI = $[0.567, 0.662]$, $p < 0.001$, $BF_{10} = 763.17$), the the ROI covering parts of superior temporal lobule and intraparietal sulcus (IPS; $ACC = 0.541$, $SE = 0.009$, 95% CI = $[0.521, 0.562]$, $p < 0.001$, $BF_{10} = 122.82$), in the ROI covering parts of right middle temporal gyrus and the lateral occipital cortex (LOC $ACC = 0.529$, $SE = 0.013$, 95% CI = $[0.501, 0.557]$, $p = 0.041$, $BF_{10} = 1.414$), but not in the ROI covering parts of the left and right supra marginal gyrus (SMG $ACC = 0.509$, $SE = 0.006$, 95% CI = $[0.495, 0.522]$, $p = 0.180$, $BF_{10} = 0.460$; see Sup. Fig. 2). Please note that even though this approach provides similar results to the main analysis, it is confounded by the low-level perceptual features of the CS stimuli.

Supplementary Tables

Supplementary Table 1 Summary of the trials per condition in one run of the Pavlovian task

	Salty Outcome		Sweet Outcome		No Snack
	Left	Right	Left	Right	
CS+ salty left	7	1	1	0	1
CS+ salty right	1	7	0	1	1
CS+ sweet left	1	0	7	1	1
CS+ sweet right	0	1	1	7	1
CS-	0	1	1	1	7
CS-	1	1	1	0	7

Note. The experiment included three runs, the last run was administered under extinction. CS+ = positive conditioned stimulus. CS- = negative conditioned stimulus.

Supplementary Figures

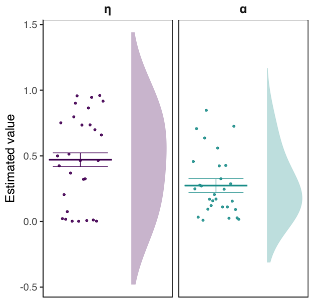

Supplementary Fig. 3 Distribution of the estimated value of the free parameters. Mean estimated value of the learning parameters for the Forward model (η) and Rescorla Wagner model(α). Error bars indicate the within–participant s.e.m. $N = 29$ participants.

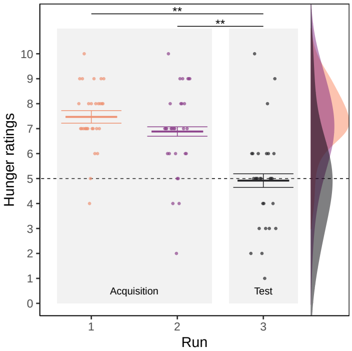

Supplementary Fig. 4 Hunger level during the experimental session. Mean level of hunger ratings over the three runs of the experimental session. The outcome devaluation procedure was administered after the acquisition session (run 1 and 2) and before the test session (run 3). Error bars indicate the within–participant s.e.m. Statistical significance was determined by the effect of run (i.e., 1 vs 2; 1 vs 3; 2 vs 3) in a linear mixed-effects model. Asterisks indicate statistically significant differences (1 vs 3; $F_{(1,28.06)} = 41.644$, $p < 0.001$; 2 vs 3 $F_{(1,27.66)} = 39.388$, $p < 0.001$). $N = 29$ participants. Double asterisks indicate statistically significant differences between conditions that survived correction for multiple comparisons. $N = 29$ participants.

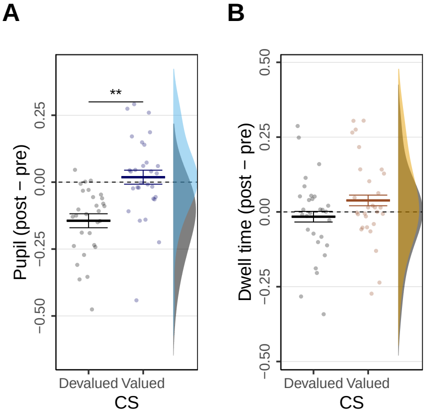

Supplementary Fig. 5 Effects of outcome devaluation on eye behavior. Mean difference of the devaluation induced changes for the CS valued and the CS devalued (post - pre devaluation) in: **(A)** the pupil response (CS- corrected) and **(B)** the dwell time of the anticipatory gaze direction (CS- corrected). Error bars indicate the within–participant s.e.m. Statistical significance was determined by the interaction term (session: pre- or post-devaluation x CS: value or devalued) in a linear mixed-effects model. Asterisks indicate statistically significant differences ($\beta = 0.040$, $SE = 0.008$, 95% CI $= [0.023, 0.057]$, $p < 0.0001$, $BF_{10} = 44.77$). $N = 29$ participants.

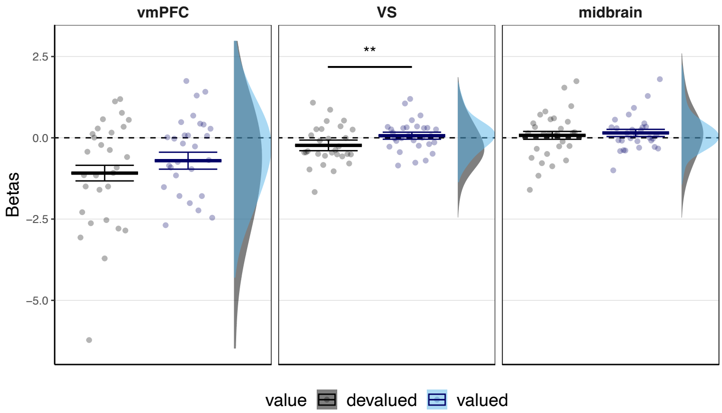

Supplementary Fig. 6 Sensitivity to outcome devaluation in Reward Prediction Error Regions of interest (ROI). Betas for the valued and the devalued contrast in the midbrain ROI, the ventral striatum / sgACC ROI (VS), and the ventromedial prefrontal cortex ROI (vmPFC). The "valued contrast" was defined as the difference in the BOLD signal during the outcome delivery (displayed behind two black patches) after the perception of the valued CS+ versus the CS-. The "devalued contrast" was defined as the difference in the BOLD signal during the outcome delivery (displayed behind two black patches) after the perception of the devalued CS+ versus the CS-. Error bars indicate the within–participant s.e.m. Statistical significance was determined by the effect of the outcome value (value or devalued) in a linear mixed model. Asterisks indicate statistically significant differences that survived correction for multiple comparisons ($\beta = -0.149$, $SE = 0.057$, 95% CI $= [-0.267, -0.030]$, $p = 0.0157$, $BF_{10} = 2.98$). $N = 29$ participants.

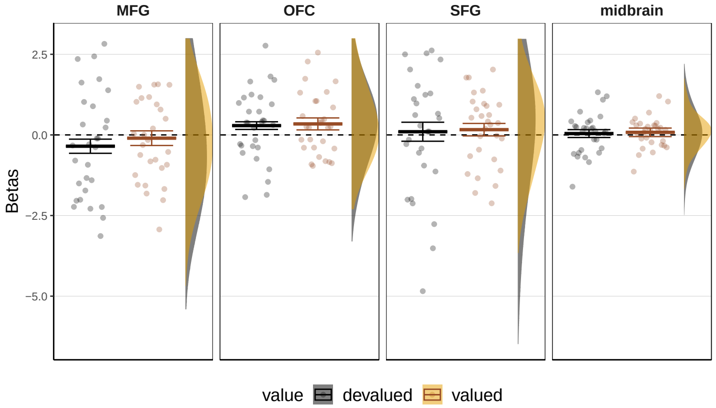

Supplementary Fig. 7 Sensitivity to outcome devaluation in State Prediction Error ROIs. Betas for the devalued and valued contrast in the midbrain ROI, the superior frontal gyrus ROI (SFG), the bilateral orbitofrontal/anterior insula ROI (OFC), and the middle prefrontal gyrus/inferior frontal gyrus ROI (MFG). The "valued contrast" was defined as the difference in the BOLD signal during the outcome delivery (displayed behind two black patches) after the perception of the CS+ valued versus the CS-. The "devalued contrast" was defined as the difference in the BOLD signal during the outcome delivery (displayed behind two black patches) after the perception of the CS+ devalued versus the CS-. Error bars indicate the within–participant s.e.m. $N = 29$ participants.

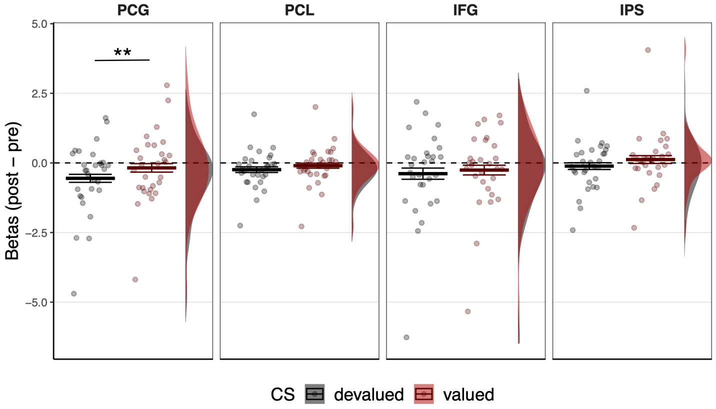

Supplementary Fig. 8 Sensitivity to outcome devaluation in ROIs decoding predictions about the taste identity representations of the outcome. Betas for the CS valued and the CS devalued (post - pre devaluation) in the ROIs identified with the MPVA analysis that decode predicted taste identity of the outcome. Error bars indicate the within–participant s.e.m. Statistical significance was determined by the effect of CS value: value or devalued in a linear mixed-effects model. Asterisks indicate statistically significant differences. Double asterisks indicate statistically significant differences that survived correction for multiple comparisons across ROIs ($\beta = -0, 187$, $SE = 0.061$, 95% CI = $[-0.313, -0.061]$, $p = 0.005$, $BF_{10} = 4.73$). $N = 29$ participants. IFG = inferior frontal gyrus; IPS = intraparietal solcus; PCL = paracentral lobule; PCG = post central gyrus.

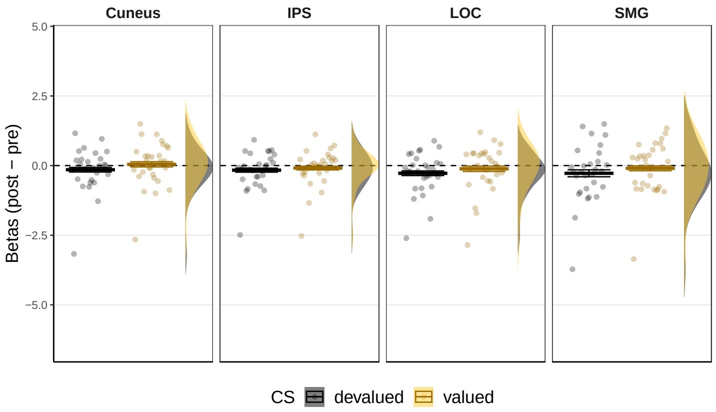

Supplementary Fig. 9 Sensitivity to outcome devaluation in ROIs decoding predictions about the location (side) representations of the outcome. Betas for the CS valued and the CS devalued (post - pre devaluation) in the ROIs identified with the MPVA analysis that decode the predicted location of the outcome delivery. Error bars indicate the within–participant s.e.m. $N = 29$ participants. IPS = intraparietal sulcus; SMG = supra marginal gyrus; LOC = Lateral occipital complex.
