# Supplementary material for: Neural substrates of parallel devaluation-sensitive and devaluation-insensitive Pavlovian learning in humans
Source: Nat Commun. 2023 Dec 5;14:8057. doi: 10.1038/s41467-023-43747-5 (PMC10697955; doi:10.1038/s41467-023-43747-5)
Supplement: Supplementary file 3 — Reporting Summary [file 41467_2023_43747_MOESM3_ESM.pdf]

nature portfolio

Corresponding author(s): Eva R. Pool

Last updated by author(s): Oct 12, 2023

Reporting Summary

Nature Portfolio wishes to improve the reproducibility of the work that we publish. This form provides structure for consistency and transparency in reporting. For further information on Nature Portfolio policies, see our Editorial Policies and the Editorial Policy Checklist.

Statistics

For all statistical analyses, confirm that the following items are present in the figure legend, table legend, main text, or Methods section.

n/a	Confirmed	
[ ]	[x]	The exact sample size (n) for each experimental group/condition, given as a discrete number and unit of measurement
[ ]	[x]	A statement on whether measurements were taken from distinct samples or whether the same sample was measured repeatedly
[ ]	[x]	The statistical test(s) used AND whether they are one- or two-sided<br>*Only common tests should be described solely by name; describe more complex techniques in the Methods section.*
[ ]	[x]	A description of all covariates tested
[ ]	[x]	A description of any assumptions or corrections, such as tests of normality and adjustment for multiple comparisons
[ ]	[x]	A full description of the statistical parameters including central tendency (e.g. means) or other basic estimates (e.g. regression coefficient) AND variation (e.g. standard deviation) or associated estimates of uncertainty (e.g. confidence intervals)
[ ]	[x]	For null hypothesis testing, the test statistic (e.g. F, t, r) with confidence intervals, effect sizes, degrees of freedom and P value noted<br>*Give P values as exact values whenever suitable.*
[ ]	[x]	For Bayesian analysis, information on the choice of priors and Markov chain Monte Carlo settings
[ ]	[x]	For hierarchical and complex designs, identification of the appropriate level for tests and full reporting of outcomes
[x]	[ ]	Estimates of effect sizes (e.g. Cohen's d, Pearson's r), indicating how they were calculated

Our web collection on statistics for biologists contains articles on many of the points above.

Software and code

Policy information about availability of computer code

Data collection	Behavioural data were collected using Psychtoolbox 3.0 implemented on Matlab (version 8.6; The Mathworks Inc., Natick, MA, USA). Eye tracking data were collected with an EyeLink 1000 Plus eye tracker. fMRI data were collected using a 3-Tesla MRI system (Magnetom Tim Trio, Siemens Medical Solutions) using a 32-channel radio frequency coil.
Data analysis	Statistical analyses of the behavioral data were performed with R (version 4.0; R Core Team, 2019). fMRI analysis were analysed using: Functional Magnetic Resonance Imaging of the Brain (FMRIB) Software Library (FSL, version 4.1), the Advanced Normalization Tools (ANTS, version 2.1), The Statistical Parametric Mapping software (SPM; version 12), the the Analysis of Functional magnetic resonance NeuroImages software (AFNI; version 20.2), and PyMVPA (version 2.5.0). Custom code developed for data analysis can be found here: https://github.com/evapool/PavlovianPredictions/

For manuscripts utilizing custom algorithms or software that are central to the research but not yet described in published literature, software must be made available to editors and reviewers. We strongly encourage code deposition in a community repository (e.g. GitHub). See the Nature Portfolio guidelines for submitting code & software for further information.

Data

Policy information about availability of data

All manuscripts must include a data availability statement. This statement should provide the following information, where applicable:

- Accession codes, unique identifiers, or web links for publicly available datasets
- A description of any restrictions on data availability
- For clinical datasets or third party data, please ensure that the statement adheres to our policy

the fMRI data generated in this study have been deposited in the YARETA database under accession code: https://doi.org/10.26037/yareta:dyhmmxkwkfbwvaq4yotxziszva

Research involving human participants, their data, or biological material

Policy information about studies with human participants or human data. See also policy information about sex, gender (identity/presentation), and sexual orientation and race, ethnicity and racism.

Reporting on sex and gender	The sample was composed of 29 participants (11 females; 18 males). Gender was self reported. No gender analysis was performed, since this was not the focus of this study.
Reporting on race, ethnicity, or other socially relevant groupings	-
Population characteristics	The sample had a mean age of 24 years (SD = 8.4 years)
Recruitment	Participants were recruited through flyers posted on campus and librarie. Participants were prescreened to ensure they were not dieting and they were asked not to eat for at least 6 h before the experimental session (they were allowed to drink water). We are not able to quantify the of the impact of potential self-selection biases given the selection criteria.
Ethics oversight	The study protocol was approved by the Human Subjects Protection committee of the California Institute of Technology (Pasadena, CA). Written informed consent was obtained from all participants. Participants received a compensation of 50 USD for their participation in this study.

Note that full information on the approval of the study protocol must also be provided in the manuscript.

Field-specific reporting

Please select the one below that is the best fit for your research. If you are not sure, read the appropriate sections before making your selection.

☒ Life sciences ☐ Behavioural & social sciences ☐ Ecological, evolutionary & environmental sciences

For a reference copy of the document with all sections, see nature.com/documents/nr-reporting-summary-flat.pdf

Life sciences study design

All studies must disclose on these points even when the disclosure is negative.

Sample size	The sample size was determined based on the smallest devaluation effect in the pupil we found in a series of previous studies using the similar behavioral paradigms (Pool et al., 2019)
Data exclusions	One participant had to be excluded from analysis because of a hardware failure during data acqui- sition.
Replication	The behavioral findings reported here replicate the findings of Experiment 2 of Pool et al., (2019). The present experiment was run only one time, without others replications attempts.
Randomization	Allocation to experimental conditions was either randomized or counterbalanced .
Blinding	Blinding was not possible: The experimenter administered the devaluation procedure and therefore was aware of the outcome stimulus that was being devalued

Reporting for specific materials, systems and methods

We require information from authors about some types of materials, experimental systems and methods used in many studies. Here, indicate whether each material, system or method listed is relevant to your study. If you are not sure if a list item applies to your research, read the appropriate section before selecting a response.

Materials & experimental systems

n/a	Involved in the study
☒	☐ Antibodies
☒	☐ Eukaryotic cell lines
☒	☐ Palaeontology and archaeology
☒	☐ Animals and other organisms
☒	☐ Clinical data
☒	☐ Dual use research of concern
☒	☐ Plants

Methods

n/a	Involved in the study
☒	☐ ChIP-seq
☒	☐ Flow cytometry
☐	☒ MRI-based neuroimaging

Magnetic resonance imaging

Experimental design

Design type

Event-related design

Design specifications

The task consisted of three runs lasting approximately 15 min each. Each run was composed of 60 trials for a total of 180 trials.
Each trial was composed of (a) a cue presented for 1.5 s to 4.5 s in either the upper or lower white frames; (b) an empty screen with only the background white frames presented for 3 s, and (c) a video of the experimenter's hand delivering their favorite snack into a small bag lasting 3 s. When the video appeared in either the left or the right white frame (see Fig 1) a picture depicting the small bag without any action was displayed on the opposite side of the screen. If no video was displayed, both sides displayed a picture of the small bag without any action.
The inter-trial interval consisted of a fixation cross and was presented for 4 s to 8 s (uniformly distributed).

Behavioral performance measures

There were 3 behavioral measures during the task: reaction times during a button press task, Pupil dilation and Gaze direction.

Acquisition

Imaging type(s)

functional and anatomical

Field strength

3T

Sequence & imaging parameters

Functional images were acquired using a multi-band echo-planar imaging (EPI) sequence with the following parameters: 56 axial slices (whole-brain), A-P phase encoding, echo time (TE) = 30 ms, repetition time (TR) = 1000 ms, multi band acceleration of 4, field of view (FoV) = 200 × 200 mm, flip angle = 60◦, 2.5 mm isotropic resolution, EPI factor of 80, echo spacing = 0.54 ms. Positive and negative polarity EPI- based field maps were collected before each run to allow geometric correction of the EPI data. Field maps were single band, TE = 50 ms, TR = 4800 ms, flip angle = 90◦. We also acquired whole brain T1-weighted (T1w) and T2- weighted (T2w) anatomical images both with sagittal orientation (isotropic voxel size = 0.9 mm; FoV = 256 × 256 mm)

Area of acquisition

whole brain

Diffusion MRI ☐ Used ☒ Not used

Preprocessing

Preprocessing software

For the preprocessing, we combined the Functional Magnetic Resonance Imaging of the Brain (FMRIB) Software Library (FSL, version 4.1) with the Advanced Normalization Tools (ANTS, version 2.1) .
We reorientated and brain extracted all scans using fslreorient2std and the bet FSL commands, respectively. Following alignment of the T2 to the T1 (FSL flirt command).
We applied field maps to correct geometric distortions. Field maps were extracted using FSL topup. De-noised functional scans were then unwarped with field maps using FSL fugue.
we applied a spatial smoothing of 8 mm full-width half maximum (FWHM).

Normalization

We used ANTS to implement diffeomorphic co-registration of the preprocessed functional and structural images in the Montreal Neurological Institute (MNI) space, using the nearest-neighbor interpolation and leaving the functional images in their native resolution.

Normalization template

CIT168 high resolution T1 and T2 templates

Noise and artifact removal

We used an fMRI independent component analysis (ICA) to remove artifacts. The multivariate exploratory linear optimized decomposition tool (MELODIC) decomposes the raw BOLD signal into independent components (IC). These components were classified as signal or noise using a classifier that was trained on previous datasets from the laboratory. Noise components were removed from the signal using the FSL's ICA-based X-noiseifier (FIX).

Volume censoring	volume censoring was not performed

Statistical modeling & inference

Model type and settings	We used a massive univariate analysis using the regressors derived from classical reinforcement learning model and a multivariate analysis for the whole brain analysis. We used linear mixed models (lmer) to test the devaluation effects on the betas extracted from the ROIs and the behavioral data.
Effect(s) tested	We tested the neural correlates of of state and reward prediction errors, the neural correlates of multiple aspects of the outcome representation upon the conditioned stimulus perception. The sensitivity to outcome devaluation of the brain regions identified with the aforementioned tests and the sensitivity to devaluation of the behavioral measures.

Specify type of analysis: ☐ Whole brain ☐ ROI-based ☒ Both

Anatomical location(s): ROI were defined based on functional activations

Statistic type for inference (See Eklund et al. 2016)	cluster-wise
Correction	The multiple comparisons correction was done using the Analysis of Functional magnetic resonance NeuroImages software (AFNI; version 20.2). We used the 3dFWHMx function to estimate the intrinsic spatial smoothness of each dimension. Then, we used the new 3dClustSim function [85] to create – via Monte Carlo simulation to form those estimate – a cluster extent threshold corrected for multiple comparisons at $p < 0.05$ for a height threshold of $p < 0.001$ within the whole brain.

Models & analysis

n/a	Involved in the study
☐	☒ Functional and/or effective connectivity
☒	☐ Graph analysis
☐	☒ Multivariate modeling or predictive analysis

Functional and/or effective connectivity	does not apply
Multivariate modeling and predictive analysis	For the multivariate modeling we used the T-maps extracted based on the univariate analysis. Classifier training and testing was done in a cross-validated manner with 2 folds (or 3 folds) and classification analyses were performed with a linear support vector machine (SVM) classifier. We performed a whole brain searchlight analysis with a spherical searchlight, using a radius of 3 voxels. The SVM cost/penalty parameter C was set to 1.0 for all searchlight analyses. The classification accuracy of each searchlight was assigned to the center voxel of the sphere.
